# Retraction: MicroRNA-10b Promotes Nucleus Pulposus Cell Proliferation through RhoC-Akt Pathway by Targeting HOXD10 in Intervetebral Disc Degeneration

**DOI:** 10.1371/journal.pone.0283113

**Published:** 2023-03-29

**Authors:** The PLOS ONE Editors

The *PLOS ONE* Editors retract this article [1] because it was identified as one of a series of submissions for which we have concerns about peer review, authorship, and similarities across articles. Image similarities were noted between Fig 2C in [1] and Figs 3B and 7B in [2]. These concerns call into question the validity and provenance of the reported results. We regret that the issues were not addressed prior to the article’s publication.

JS and GQ agreed with the retraction. ZL, JL, and XW either did not respond directly or could not be reached. XY and WKKW responded but expressed neither agreement nor disagreement with the editorial decision.

At the time of retraction, the article [1] was republished to remove Table 1 as it was not in compliance with PLOS Human Subjects Research policy.
